# Three Supernumerary Marker Chromosomes in a Patient with Developmental Delay, Mental Retardation, and Dysmorphic Features

**DOI:** 10.4061/2011/185271

**Published:** 2011-07-17

**Authors:** Jie Hu, Suneeta Madan-Khetarpal, Alvaro H. Serrano Russi, Sally Kochmar, Stephanie J. DeWard, Malini Sathanoori, Urvashi Surti

**Affiliations:** ^1^Pittsburgh Cytogenetics Laboratory, Magee-Womens Hospital of UPMC and Department of Obstetrics, Gynecology & Reproductive Sciences, University of Pittsburgh School of Medicine, Pittsburgh, PA 15213, USA; ^2^Department of Pediatrics, University of Pittsburgh School of Medicine and Children's Hospital of Pittsburgh of UPMC, Pittsburgh, PA 15213, USA; ^3^Pittsburgh Cytogenetics Laboratory, Magee-Womens Hospital of UPMC, Pittsburgh, PA 15213, USA; ^4^Pittsburgh Cytogenetics Laboratory, Magee-Womens Hospital of UPMC and Department of Pathology, University of Pittsburgh School of Medicine, Pittsburgh, PA 15213, USA

## Abstract

We characterized three supernumerary marker chromosomes (SMCs) simultaneously present in a 2-year- and 10-month-old male patient with mental retardation and dysmorphic features. Peripheral blood chromosome analysis revealed two to three SMCs in 25/26 cells analyzed. The remaining one cell had one SMC. Microarray comparative genomic hybridization (aCGH) showed mosaicism for gains of 5q35.3, 15q11.2q13.3, and 18p11.21q11.1 regions. All three gains contain multiple OMIM genes. FISH studies indicated that one of the SMCs is a dicentric ring 15 with two copies of the 15q11.2q13.3 region including SNRPN/UBE3A and two copies of the 5q35.3 region. One of the der(18)s contains the 18 centromere and 18p11.2 regions, while the other der(18) has a signal for the 18 centromere only. The phenotype of the patient is compared with that of patients with tetrasomy 15q11.2q13.3, trisomy 5q35.3, and trisomy 18p11.2. Our study demonstrates that aCGH and FISH analyses are powerful tools, which complement the conventional cytogenetic analysis for the identification of SMCs.

## 1. Introduction

Supernumerary marker chromosomes (SMCs) are small extra abnormal chromosomes with an unknown chromosome origin detected by conventional cytogenetic analysis. SMCs are estimated to occur in 0.04% *∼* 0.05% of live births [1, 2]. The majority of SMCs are derived from acrocentric chromosomes with satellited or bisatellited constriction, and about half of them are derived from chromosome 15 [3]. In general, if the marker chromosome contains only heterochromatin, it does not result in any phenotypic abnormalities. However, 30% of the marker chromosomes contain not only heterochromatin (alpha-satellite DNA), but also euchromatin, which leads to a segmental trisomy or tetrasomy and consequent congenital anomalies [4]. The occurrence of SMCs is seven times more prevalent in individuals with mental retardation [2].

 Molecular cytogenetic techniques including fluorescence in situ hybridization (FISH) and spectral karyotyping (SKY; multicolor banding) have been used as complementary cytogenetic tools to identify the origin of SMCs. Although SKY using 24 chromosome-specific paint probes can be used for identification of chromosomal origin of an SMC, it is unable to precisely identify the DNA components of the SMC [5]. With prior knowledge of chromosomal origin of the SMC, multicolor banding can be utilized to analyze an SMC at ~10 Mb resolution [6]. In addition, FISH using specific probes can be applied to further define the SMC at the gene/loci level. However, it is time consuming and labor intensive and also requires prior knowledge of the chromosomal region. More recently, microarray comparative genomic hybridization (aCGH) has been applied to define the critical regions for gain or loss of copy numbers [7] and to further analyze complex chromosome rearrangements as well as SMCs [8]. This technique can precisely detect the DNA copy number gain or loss at the oligonucleotide level. This technique alone, however, cannot distinguish whether the copy number gain is due to tandem duplication, insertion, unbalanced rearrangement, or presence of an extra SMC. In this paper, we demonstrate the applications of aCGH as a complementary method to the standard cytogenetic and FISH analyses for the identification of three marker chromosomes in a child with developmental delay, mental retardation, and dysmorphic features.

## 2. Material and Methods

### 2.1. Clinical History

 The patient is a 2-year- and 10-month-old African-American boy. He was born at 42 weeks of gestation to a 16-year-old healthy primigravida via vaginal delivery. The pregnancy was complicated by preeclampsia. His birth weight was 7 pounds and 15 ounces, and his birth length was 21 inches. Neonatal history was unremarkable. He walked at 15 months, but his language development was severely delayed. At age of 2 years and 8 months, he used 12–15 single words. He was also hyperactive. 

 On his most recent examination at 2 years and 8 months, his height and weight were at the 75th to 95th percentile. His head circumference was at the 25th to 50th percentile. There was a mottled hypopigmentation of the skin but no whorl-shaped lesions or marble cake appearance on his skin. He had plagiocephaly with a prominent forehead and slight flattening of his occiput. His ears were borderline low set with slight posterior rotation, pointed helices, and thick cartilage. There was a small sinus dimple on the root of the helix of the right ear. He had a thin upper lip with long philtrum at 1.8 cm in length and a prominent nasal root. He also had wide-spaced nipples with an internipple distance of 13.5 cm (75th to 97th percentile). His hands measured 10 cm bilaterally (3rd to 25th percentile), and the length of his third finger was 4.3 cm bilaterally (25th percentile). He had mild syndactyly of the second and third toes.

### 2.2. Family History

 The proband's mother required an individualized education program (IEP) when she was in school. One 8-year-old cousin, a daughter of a 23-year-old maternal aunt, has developmental delay, and another 6-year-old cousin, a daughter of a 25-year-old maternal aunt, has dyslexia. His 21-year-old maternal uncle also required an IEP in school. The paternal information was not available.

### 2.3. Cytogenetics, FISH, and aCGH Studies

 High-resolution chromosome analysis was performed on the peripheral blood specimen using standard cytogenetics protocols. Array-CGH was performed at Signature Genomic Laboratories (Spokane, WA) as described elsewhere [9]. A constitutional SignatureChipOS consisting of 135,000 oligonucleotides with 3397 loci printed on the microarray was used to characterize the patient DNA. FISH was performed using BAC clones RP11-305G6 (5q35.3), CTD-2583E4 (5q35.3), RP11-1122J3 (15q11.2), D15S11(15q11.2), RP11-959E3(15q11.2-15q13.3), RP11-411B10(18p11.21), RP11-703l16(18p11.21), and centromeric probes (D15Z4, D15Z1, and D18Z1) (Vysis, Downers Grove, IL) to confirm and further analyze the aCGH results.

## 3. Results

### 3.1. Cytogenetic and aCGH Findings

**Figure 1:**
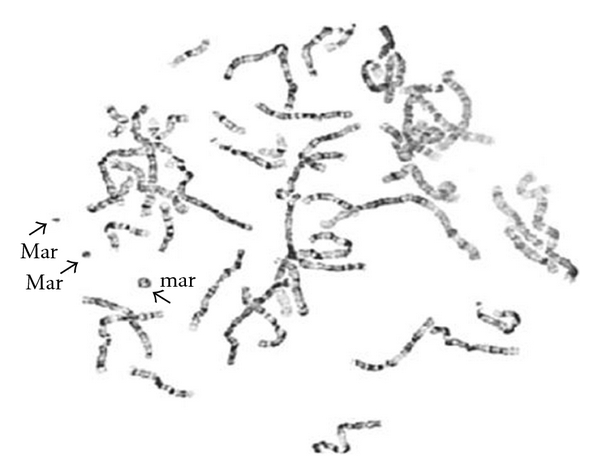
G-banded metaphase spread showing three SMCs.

**Figure 2:**
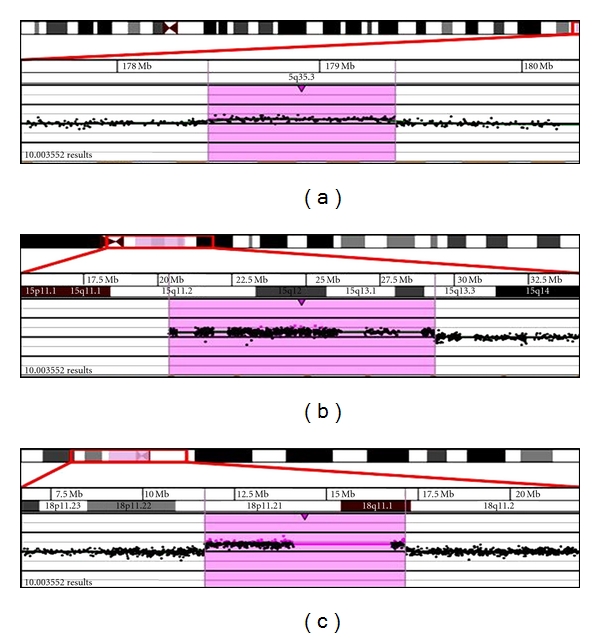
aCGH showing (a) a mosaic gain of the 5q35.3 region, (b) a mosaic gain of the 15q11.2q13.3 region, and (c) a mosaic gain of the 18p11.21q11.1 region.

**Figure 3:**
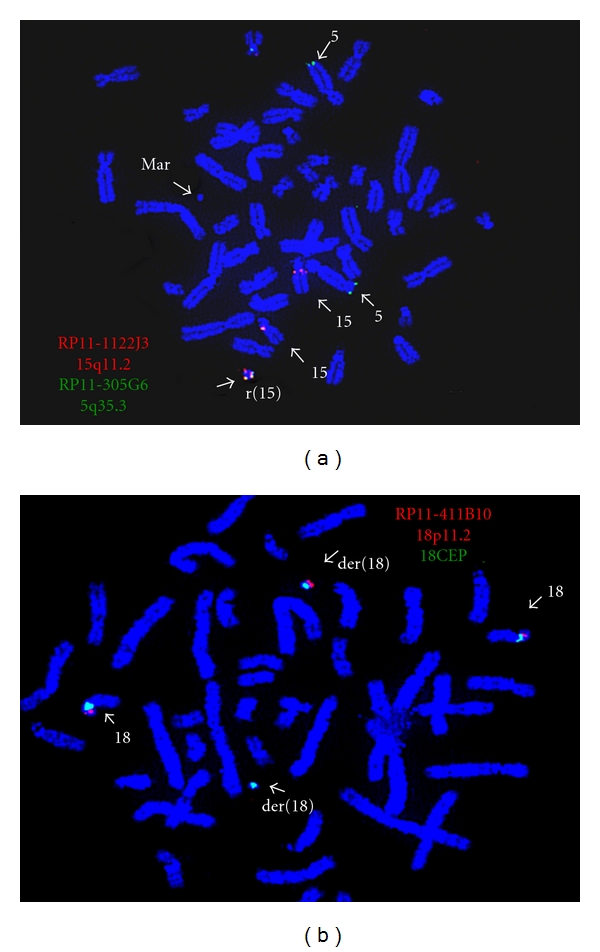
(a) FISH showing the ring chromosome containing two signals for the RP11-305G6 (green, 5q35.3) and two signals for RP11-1122J3 (red, 15q11.2); (b) FISH showing one der(18) with signal for D18Z1 (green) only and another der(18) with one signal for D18Z1 (green) and for RP11-703l16 (red, 18p11.2).

 Cytogenetic analysis of PHA-stimulated peripheral blood lymphocytes revealed a 48,XY,+2mar[16]/49,XY,+3mar,[9]/47,XY,+mar[1] chromosome pattern in 26 cells analyzed. One of the markers appeared to be a ring chromosome. A metaphase spread with three marker chromosomes is shown in Figure 1. Microarray analysis revealed mosaicism for a 1.0 Mb interstitial gain of the 5q35.3 region [arr 5q35.3(178,486,666-179,522,156x3)] (Figure 2(a)), a 9.0 Mb interstitial gain of the 15q11.2q13.3 region [arr 15q11.2q13.3(20372901-29351062x3)] (Figure 2(b)), and a 5.5 Mb mosaic interstitial gain of the 18p11.21q11.1 region [arr 18p11.21q11.1(11,690,934-17,148,187x3)] (Figure 2(c)). Metaphase FISH studies showed that the ring chromosome contained two signals for the chromosome 15 centromeric probe (D15Z4), no signals for D15Z1 probe, two signals for each of the chromosome 15q11.2q13 probes, D15S11(15q11.2), RP11-1122J3(15q11.2), and RP11-959E3(15q13), and two signals for each of the chromosome 5q35.3 region probes, CTD-2583E4 and RP11-305G6. FISH image of a metaphase cell showing the r(15) with two signals for each of the RP11-1122J3(15q11.2) and RP11-305G6 (5q35.3) probes is shown in Figure 3(a). These FISH results indicate that the ring chromosome is a dicentric r(15) with two copies of chromosome 5q35.3 inserted into the ring. The presence of this ring results in mosaic tetrasomy of the 5q35.3 and 15q11.2q13.3 regions. The second and third marker chromosomes are derivative chromosome 18s. One of the der(18)s contained one signal for each of the D18Z1 probe (18CEP) and the 18p11.2 probes (RP11-411B10, RP11-703l16), while the other der(18) contained a signal for the D18Z1(18CEP) only. The presence of these der(18)s results in segmental trisomy for the chromosome 18p11.21 region. Figure 3(b) shows a metaphase cell with two der(18)s. The FISH karyotype for the markers is described as follows: ish dic r(15)ins(15;5) (?;q35.3q35.3)(D15Z4++, D15Z1−, D15S11++, RP11-1122J3++, RP11-959E3++, RP11-305G6++, CTD-2583E4++), der(18)(D18Z1+, RP11-703l16+, RP11-411B10+), der(18)(D18Z1+, RP11-703l16−, RP11-411B10−).

 To estimate the levels of the mosaicism for each marker one hundred interphase cells were counted for each set of probes. The analysis revealed 20.2% interphase cells with r(15), 50.5% interphase cells with both der(18)s, 29.7% interphase cells with the der(18) containing centromere and 18p11.2 region, and 16.8% interphase cells with the der(18) containing 18 centromeric region only. Only three percent of cells analyzed showed a normal hybridization pattern for the chromosome 18CEP and 18p probes. 

 Maternal peripheral blood chromosome analysis is normal 46,XX in all cells studied. Paternal peripheral blood sample was not available for current study. 

## 4. Discussion

In recent years, aCGH has been increasingly utilized for genetic testing of individuals with idiopathic mental retardation, developmental delay, autism spectrum disorders, and multiple congenital anomalies. By combining the aCGH technique with the classical cytogenetic and FISH analyses, we are able to identify cryptic genomic alterations and to further analyze gross genomic alterations identified by the classical cytogenetic analysis. Individual segmental trisomies or tetrasomies 5q35.3, 15q112q13.3, and 18p11.2 resulting from an SMC were reported in patients with varying degrees of mental retardation [10–16]. However, simultaneous occurrence of the trisomies or tetrasomies of 5q35.3, 15q11.2q13.3, and 18p11.21 has not been reported in the literature. 

 The tetrasomic region of 15q11.2q13.3 detected in our patient contains 26 OMIM genes (*TUBGCP5, CYFIP1, NIPA2, NIPA1, MKRN3, MAGEL2, NDN, PWRN2, PWRN1, C15orf2, SNRPN, PAR5, IPW, PAR1, UBE3A, ATP10A, GABRB3, GABRA5, GABRG3, OCA2, HERC2, APBA2, NDNL2, TJP1, CHRFAM7A, and TRPM1*). This region is highly susceptible to genomic alterations, including interstitial deletions, duplications, triplications, inversions, and the formation of SMCs. There are three well-known syndromes associated with this region: Prader Willi syndrome (PWS), Angelman syndrome (AS), and 15q13.3 duplication syndrome. 

 Increase in copies of the genes in this region can occur as a result of interstitial duplications and triplications or as a result of SMCs [17–23]. Individuals with 15q duplication syndrome commonly have hypotonia, developmental delay, learning disabilities, autism spectrum disorders (ASDs), epilepsy, and characteristic facial features [24]. The phenotypes vary significantly. Some individuals also have anxiety, hyperactivity, and short stature [25]. The variability in phenotype appears to be influenced by the nature of the alteration, the parental origin of the alteration, and the level of mosaicism [26]. It was thought that patients with maternally inherited dup 15q11q13 have more severe neurobehavioral phenotype, which often includes moderate-to-severe mental retardation, seizures, poor motor coordination, autistic behavior, and mild dysmorphic features [27–30]. These may result from the increased expression of maternally expressed dosage-sensitive genes, such as *UBE3A*. Patients with paternally derived duplication of 15q11q13 are often associated with severe abnormal phenotype, including marked developmental delay, ASD, and behavior problems [23, 31–34]. A recent study in a large family with 12 carriers in three generations suggested a possible reduced penetrance in a duplication of paternal origin in that family [27]. 

 In addition to the parental origin, the dosage of the Prader Willi/Angelman critical region (PWACR) is a major factor contributing to the clinical severity [35–37]. Mosaicisms of maternally derived SMC 15 and paternally derived SMC 15 have been reported [3, 38, 39]. Overall, 28% of the acrocentric SMCs and 69% of nonacrocentric SMCs were found in association with a normal cell line [3]. Clinical observations and animal studies have shown that the phenotype associated with tetrasomy and hexasomy of the PWACR is significantly more severe than that associated with trisomy [40–43]. 

 Although our patient has a tetrasomy for the 15q11.2q13.3 region, only a few mild phenotypic features associated with duplication or triplication of 15q11.13 are present in our patient, including delayed development, speech delay, hyperactivity, and downslanted palpebral fissures. The mild phenotype may be due to the mosaicism. We were unable to determine the parental origin of the ring 15. 

 The copy number gain of the 5q35.3 region in our patient contains 8 OMIM genes (*ADAMTS2, RUFY1, HNRNPH1, CANX, MAML1, LTC4S, MGAT4B,* and *SQSTM1*). Segmental trisomy or tetrasomy for 5q35.2q35.3 is rare. There are only a few cases with pure segmental trisomy for 5q35.2q35.3 reported in the literature [10, 11], and segmental tetrasomy of this region has not been reported. Patients with segmental trisomy for 5q35.2q35.3 have clinical features of developmental delay, motor retardation, speech delay, mental retardation, as well as brachydactyly, thin upper lip, and craniosynostosis [10, 11] (Table 1). Our patient has all of these clinical features. The critical genes associated with the phenotype of the 5q35.2 q35.3 duplication have not been identified. 

 Trisomy 18p has been reported in the literature. Most of the cases are due to unbalanced translocation [44]. Trisomy 18p as a result of SMC has only been reported in a handful of cases [16, 44, 45]. The most common phenotypic association is of minor facial anomaly and subnormal mental development [45]. The limited number of reported cases with SMC 18 may be due to the unavailability of suitable techniques in the past. The clinical application of aCGH for the identification of SMC will be very useful for identification of marker chromosomes. The 18p11.2.1 region with gain of copy number for our patient contains 15 OMIM genes *(GNAL, CHMP1B, MPPE1, IMPA2, CIDEA, AFG3L2, SPIRE1, PSMG2, PTPN2, SEH1L, C18orf1, RNMT, MC5R, MC2R, and ROCK1).*

 Because of the simultaneous presence of three marker chromosomes, our patient has segmental tetrasomies of 5q35.3 and 15q11.2q13.3 and a segmental trisomy of 18p11.2 in a mosaic status. He has clinical features of each of the three syndromes. The mottled hypopigmentation observed on the patient's skin may be associated with the chromosome mosaicism. However, the skin biopsy on the area with hypopigmentation was not performed. This is the first case report which characterizes the origin and gene content of three distinct SMCs derived from three different chromosomes presenting in a mosaic status in a patient with developmental delay, mental retardation, and dysmorphic features. Understanding the origin of the SMC and the gain or loss of genes in any given chromosome abnormality is an important step to predicting the phenotypic outcome of an identified abnormality. This study demonstrated that the aCGH technique in combination with FISH analysis is a powerful tool to identify the origin of SMC.

## Figures and Tables

**Table 1:** Phenotypic comparison of the reported cases (mosaic or nonmosaic pure partial trisomy 5q35.3) with the present patient.

Authors	Hunter et al. [10]				Chen et al. [11]	Present
	Patient IV. 11	Patient IV. 5	Patient V. 13	Patient V. 14		
Duplication	q35qter	q35qter	q35qter	q35qter	q35.2q35.3	q35.3q35.3
Origin of duplication	t(5;13)(q35;p11.2)	t(5;13)(q35;p11.2)	t(5;13)(q35;p11.2)	t(5;13)(q35;p11.2)	dir dup	Marker
Birth weight (g)	NA	NA	3,230	2,325	2,100	3,500
Sex	M	F	F	F	F	M
Age at examination	31 y and 57 y	42 y and 68 y	3 y and 29 y	6.5 y and 31 y	11 y	2 y 10 M
Growth retardation	+	+	+	+	+	−
Mental retardation	+	+	+	+	+	+
Motor retardation	NA	NA	NA	NA	+	+
Speech retardation						+
Microcephaly	+	+	+	+	+	Plagiocephaly
Antimongoloid slant	−	−	−	−	+	−
Strabismus	−	−	−	−	+	−
Thin upper lip	+	+	+	+	+	+
Downturned mouth	+	+	+	+	+	
Ear anomaly	−	−	−	−	−	+
Brachydactyly	+	+	+	+	+	
Syndactyly						+ (toe)
Congenital heart defects	+	−	−	+	−	
Others				Craniosynostosis	Inguinal hernias	
